# Effects of contrast medium injection pressure on angiographic image quality

**DOI:** 10.3389/fradi.2025.1723413

**Published:** 2026-02-02

**Authors:** Sara A. Abosabie, Tabea Kao, Timo Alexander Auer, Dirk Schnapauff, Uli Fehrenbach, Federico Collettini, Martin Jonczyk, Willie Magnus Lüdemann, Charlie Alexander Hamm, Gero Wieners, Robin Schmidt, Emine Yaren Yilmaz, Yubei He, Bernhard Gebauer, Anne Frisch, Lynn Jeanette Savic

**Affiliations:** 1 Department of Radiology, Charité - Universitätsmedizin Berlin, Corporate Member of Freie Universität Berlin and Humboldt-Universität zu Berlin, Berlin, Germany; 2 Department of Radiology and Biomedical Imaging, Yale University School of Medicine, New Haven, CT, United States; 3 Berlin Institute of Health at Charité - Universitätsmedizin Berlin, Berlin, Germany; 4 Department of Radiology, Carl-Thiem-Klinikum, Cottbus, Germany; 5 Experimental Clinical Research Center (ECRC) at Charité - Universitätsmedizin Berlin and Max-Delbrück-Centrum für molekulare Medizin (MDC), Berlin, Germany

**Keywords:** digital subtraction angiography, injection pressure, intra-arterial therapies, liver cancer, microcatheter

## Abstract

**Rationale and objectives:**

To evaluate if higher contrast medium injection pressure can improve image quality of digital subtraction angiography (DSA) in liver-directed interventions.

**Materials and methods:**

Prospective single-center study including twelve patients with hepatocellular carcinoma (*n* = 11) or liver metastases (*n* = 1) undergoing intra-arterial therapies to systematically compare DSA image quality (primary endpoint) and radiation exposure (secondary endpoint) using two microcatheters with maximum application pressures 750 (“C750”) and 1,200 PSI (“C1200”). Patients underwent two DSAs with both microcatheters placed in the common hepatic artery. Application pressure, contrast medium flow, volume, and dose area product were recorded. Image quality was evaluated using a customized questionnaire and quantified by calculating signal- and contrast-to-noise-ratios, and vessel-to-liver signal intensity ratio. Results were compared by paired *t*-test and Wilcoxon Signed-Rank test.

**Results:**

Image quality using C1200 (achieved 917 ± 94 PSI) was rated more favorably than C750 (731 ± 45 PSI). C1200 reached higher scores of artery visualization in 72% cases, and significantly outperformed C750 regarding tumor blush in 100% (*p* < 0.001). Contrast-to-noise-ratio were significantly higher in C1200 (*p* < 0.001) and vessel-to-liver intensity ratios were significantly lower (*p* = 0.001), both reflecting improved visualization and delineation of liver vessels. Signal-to-noise ratio did not differ significantly. Mean dose area product was comparable (C1200; 371.9 vs. 374.8 μGym^2^).

**Conclusion:**

The results suggest benefits of standardized CM injections for DSA using higher application pressure to enhance image contrast and tumor demarcation during IAT.

## Key points

- The effect of contrast media injection pressure on image quality is unexplored.
- Higher injection pressure using 1,200 PSI microcatheter improved angiographic image quality.
- A 1,200 PSI microcatheter showed superior arterial contrast and tumor blush visualization.
- The 1,200 PSI microcatheter achieved higher contrast-to-noise ratios (*p* = 0.001) in quantitative analysis.

## 1 Introduction

Hypervascular liver tumors, including hepatocellular carcinoma (HCC) and certain liver metastases, are characterized by a richer arterial blood supply than the surrounding normal liver tissue (1). Intra-arterial therapies (IATs) such as transarterial chemoembolization (TACE) and transarterial radioembolization (TARE) are minimally-invasive catheter-based interventions that target tumor hypervascularity (2, 3). TACE is indicated for BCLC B patients with large or multinodular tumors without macrovascular invasion or extrahepatic lesions, and can be used to downstage patients for resection or transplantation (4). IATs are performed under fluoroscopy guidance where embolic and therapeutic agents are directly injected into the arteries supplying the target tumors (5).

Digital subtraction angiography (DSA) is performed to identify tumor-feeding blood vessels during IATs, providing real-time, high-contrast images and enabling precise catheter placement and embolization (6). This minimizes drug exposure to healthy tissue and optimizes therapeutic outcomes (7). Injectors can be used to deliver contrast media (CM) with constant flow rate and standardized injection pressures. Microcatheters are used to achieve super-selective catheterization of the hepatic artery (4). The microcatheters typically used for IATs have a specific maximum pressure (P_max_), commonly ranging from 750 to 800 PSI. Newer catheters are designed to tolerate higher injection pressures.

While the effect of CM injection rate on vessel enhancement during DSA has been previously studied (8), there is a lack of studies investigating the effect on DSA image quality and radiation exposure. This prospective study was designed to test the effect of a higher CM injection pressure during DSA on image quality (primary endpoint) and radiation exposure (secondary endpoint) using two microcatheters with different maximum pressure tolerance for IATs of hypervascular liver tumors. This study hypothesizes that higher CM injection pressures can improve DSA image quality by rapid and homogenous CM distribution in arteries, potentially resulting in shorter and fewer DSA acquisitions and reduced radiation exposure.

## 2 Materials and methods

### 2.1 Study cohort

This prospective clinical single-center pilot study included patients with hypervascular liver tumors meeting the eligibility criteria. Inclusion criteria were (a) 18 years or older (b) HCC or liver metastases confirmed on pretreatment MRI/CT and (c) indication for IAT (TACE or TARE) at our institution between 10/2022 and 03/2023 as determined by a multidisciplinary tumor board. Exclusion criteria were (a) hypovascular liver malignancies (b) kidney failure, and (c) known hypersensitivity reactions to iodinated contrast media [see Supplementary Appendix S1 for patient selection process according to STROBE guidelines (9)]. The study systematically compared two microcatheters with different P_max_ values: the 2.4Fr × 130 cm DraKon microcatheter (Argon Medical Devices, P_max_ 1,200 PSI) referred to as “C1200”, and the 2.4Fr × 130 cm Progreat microcatheter (Terumo, P_max_ 750 PSI), referred to as “C750”. Both are commercially available and used in IATs, differing only in P_max_. All procedures were performed in accordance with the ethical standards of the institutional and/or national research committee and with the 1964 Helsinki declaration and its later amendments or comparable ethical standards. Informed consent was obtained. This study was funded by Guerbet. The funder had no role in the study design, data acquisition, interpretation of results and writing of the manuscript.

### 2.2 Preclinical injection pressure measurements

**Figure 1:**
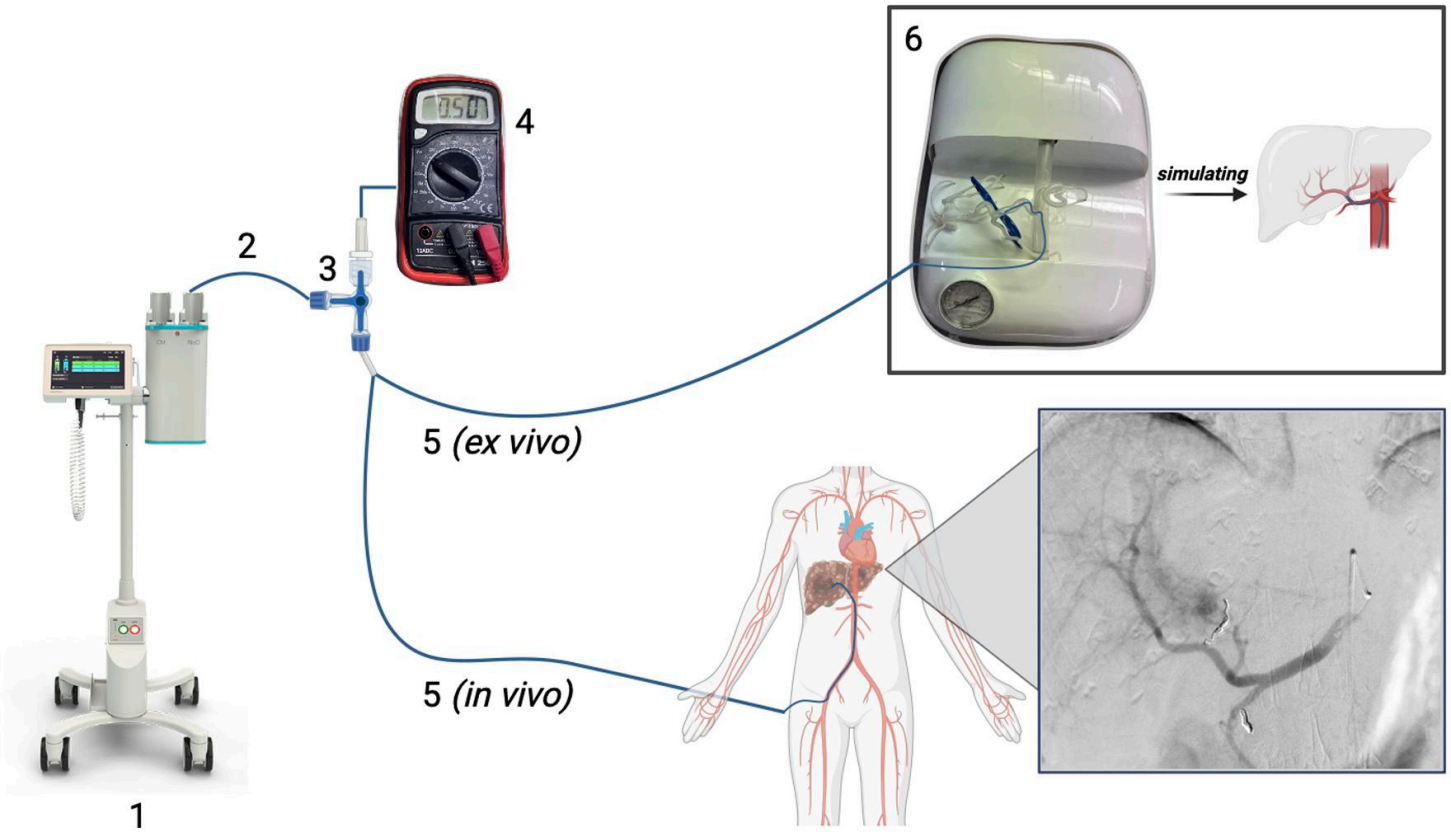
Shows the setup used to measure the pressure in the *ex vivo* angiography model (top) and during the IAT (bottom). The piston of the Accutron HP CM injector (1, Medtron AG) is filled with contrast media. The FLEX120RAN high-pressure extension tube (2, Guerbet) connects the CM injector and a high-pressure L306 three-way stopcock (3, BD). The three-way stopcock is connected to the ME780S pressure sensor (4, Amsys) and the C1200 (Argon Medical Devices) or C750 (Terumo) microcatheter (5) which feeds into the hepatic angiography model (6, Guerbet) in the *ex vivo* setup. The microcatheter is placed in the common hepatic artery by the interventional radiologist under fluoroscopy in the *in vivo* setup (bottom).

To achieve the desired injection pressure, a preclinical testing set-up was implemented. The CM injector (Medtron AG), pressure sensor (ME780S, Amsys) and microcatheter were connected via a three-way stopcock. The sensor measured CM pressure immediately before entering the microcatheter (Figure 1; Supplementary Appendix S2). A custom *ex vivo* hepatic angiography model was used to test injection settings targeting 1,200 PSI (Supplementary Appendix S3). Injection pressure was monitored using (a) the time-pressure curve displayed on the injector screen, and (b) the pressure reading from the pressure sensor (Supplementary Appendix S4). As pressure was measured in two different locations (injector vs. microcatheter), pressure readings between the two devices used (CM injector vs. sensor) were expected to differ.

### 2.3 Clinical setup and DSA

#### 2.3.1 Setup

Identical to the *ex vivo* setup, the injector was connected to the extension tube, the pressure sensor, and microcatheter (Figure 1). The injector was set to inject 20 mL of CM at 4 mL/s. The pressure limits on the injector were 750 and 1,200 PSI for C750 and C1200, respectively. The injector terminated the injection when the set pressure limit was reached regardless of the volume of CM injected. The sensor measured the injection pressure when CM entered the microcatheter, reflecting the exerted pressure in the common hepatic artery (CHA) (Figure 1; Supplementary Appendix S2). The angiographic machine (ARTIS icono, Siemens) was used according to standard procedure and includes a cone-beam computed tomography (CBCT) option. The contrast media used for each patient contained 300 mg iodine/mL (Imeron 300, Bracco Imaging). Key acquisition parameters for each patient are detailed in Supplementary Appendix S8.

#### 2.3.2 Intra-arterial therapies

Two DSAs (two frames per second with a field of view of 42 cm) were performed using both C750 and C1200 placed in the CHA in all patients to ensure comparability. The order in which the catheters were used was randomized.

#### 2.3.3 Clinical intraprocedural documentation

For each CM injection, the patient's vitals, number of images per DSA series, volume of CM injected, maximum injector pressure, and maximum CM pressure were documented. The dose area product (DAP) was recorded to indicate the patient's radiation exposure during DSA.

### 2.4 Assessment of image quality

#### 2.4.1 Qualitative image assessment

Three interventional radiologists (6, 8, and 7 years of experience), independently completed a standardized 16-question image quality questionnaire, blinded to the microcatheter used to reduce bias. Qualitative imaging components were rated using a Likert scale or direct comparison and grouped into four categories: A (visualization of liver arteries, CM reflux, and flow quality), B (tumor blush), C (tumor to parenchymal contrast), D (catheter instability/dislocation). Details can be found in Supplementary Appendix S5.

#### 2.4.2 Quantitative image assessment

Contrast-to-noise-ratios (CNR), signal-to-noise-ratios (SNR), and vessel-to-liver signal intensity ratios (VLR) were calculated based on previous studies using CT angiography (10, 11). Briefly, triplicate regions of interest (ROIs) were placed in five arterial segments and non-tumoral liver parenchyma on representative DSA images for each patient by a board-certified radiologist with 7 years of experience. In DSA images, when hepatic arteries appear darker and better delineated due to contrast medium, the signal intensity values within the vessel ROIs are lower. As a result, both the signal-to-noise ratio (SNR) and vessel-to-liver signal intensity ratio (VLR) are expected to be lower in better delineated vessels. In contrast, the contrast-to-noise ratio (CNR) is expected to be higher, reflecting the increased difference in signal intensity between the vessels and the surrounding liver parenchyma. The quantitative metric calculation is outlined in Supplementary Appendix S6.

### 2.5 Statistical analysis

To limit radiation exposure for patients during DSA, the study was designed as a pilot study with the support of a statistician, eliminating the need for a formal sample size calculation (12). A sample size of *n* = 12 is generally considered adequate for pilot studies (13). With this sample, a power analysis based on existing DSA data indicated that a mean difference of 0.55 could be detected with 82% power and a 5% alpha level, assuming an effect size of 0.92 for a two-tailed matched-pair comparison. The Wilcoxon Signed-Rank test was used to compare DAP, CM volume injected and mean pressure reached with each catheter, as well as SNR, CNR and VLR. Paired *t*-tests were performed to compare scores in each questionnaire category. Intraclass Correlation Coefficient (ICC) was evaluated to assess interrater agreement. Data was analyzed using SPSS (version 29.0, IBM Corp). Statistical significance was defined as *p* < 0.05.

## 3 Results

### 3.1 Study cohort

The study cohort consisted of twelve patients, including 7 men and 5 women (mean age 68 ± 12.5). Eleven patients with the diagnosis HCC and one patient with uveal melanoma liver metastases were evaluated. TACE (*n* = 8) and TARE (*n* = 4) procedures were performed at our institution between October 2022 and March 2023. Patient demographics are shown in Table 1.

**Table 1:** Shows the patient demographics.

Characteristics	*N* = 12 (%)
Age	
Mean ± SD	68 ± 12.5
Sex	
Male	7 (58)
Female	5 (42)
Diagnosis	
HCC	11 (91)
Uveal melanoma liver metastasis	1 (9)
Cirrhosis	
Yes	11 (91)
No	1 (9)
Etiology of cirrhosis	
Hepatitis B Virus (HBV)	2 (18)
Hepatitis C Virus (HCV)	2 (18)
Alcohol-related	4 (36)
Not specified	3 (27)
Child-Pugh Class	
A	9 (82)
B	2 (18)
C	0 (0)
MELD score	
≤10	8 (67)
≥10	3 (25)
Not applicable	1 (8)
BCLC	
A	3 (27)
B	5 (45)
C	3 (27)
D	0 (0)
Treatment	
TACE	8 (67)
TARE	4 (33)

HCC, hepatocellular carcinoma; MELD, model for end-stage liver disease; BCLC, barcelona clinic liver cancer; TACE, transarterial chemoembolization; TARE, transarterial radioembolization.

### 3.2 Preclinical injection pressure measurement

The *ex vivo* setup was feasible. The injector settings of 20 mL CM at 4 mL/s achieved 1,200 PSI injection pressure in all patients.

### 3.3 Clinical setup and DSA

All *in vivo* measurements were technically successful with both C750 and C1200 used once per patient. Injection pressure measured by the pressure sensor was higher using C1200 (mean ± SD: 1,139 ± 363 PSI) than C750 (mean ± SD: 896 ± 280 PSI), but not statistically significant (*p* = 0.09). In contrast, injector-recorded pressure was significantly higher for C1200 (mean ± SD: 917 ± 94 PSI) than C750 (mean ± SD: 731 ± 45 PSI) (*p* < 0.0001). In 10/12 patients with C750, the injector halted injection upon exceeding 750 PSI, preventing full CM delivery. In contrast, C1200 delivered the full 20 mL CM in all cases (mean ± SD: 20.0 ± 0.0 vs. 17.7 ± 3.0 mL, *p* = 0.01; see Supplementary Appendix S7 for per-patient volumes). DSA series with C1200 had a slightly lower DAP than C750 (mean ± SD: 371.9 ± 191.2 vs. 374.8 ± 242.3 µGym^2^, *p* = 0.95), but this result was not significant. The mean number of DSA images per series was similar (21.7 and 21.8 images for C750 and C1200, respectively; see Supplementary Appendix S9).

### 3.4 Assessment of image quality

#### 3.4.1 Qualitative image assessment

Across all categories, C1200 was rated more favorably than C750 by all readers (Supplementary Appendix S10). The total score of C1200 was higher than C750 in 69% of cases (mean score: 15 vs. 11 points, *p* = 0.13). ICC showed moderate interrater agreement (αC1200: 0.71 (95% confidence interval (CI): [0.24, 0.91], *p* = 0.006, αC750: 0.70 [95% CI: (0.20, 0.91), *p* = 0.008]. ICC values up to 0.75 are considered “moderate agreement” (14), but the *p*-value denotes significant reliability (*p* = 0.006 and 0.008).

For liver artery visualization (category A), C1200 outperformed C750 in 67% (mean score: 14.8 vs. 12 points, *p* = 0.22), 50% (mean score: 13 vs. 12.5 points *p* = 0.87), and 100% (mean score: 12.3 vs. 8.3 points, *p* = 0.03) of patients for readers 1–3, respectively (Table 2). Only reader 3 observed a statistically significant difference of liver artery visualization (*p* = 0.03, Table 2). The number of patients with visible reflux was small and comparable between both catheters (C750: 4 vs. C1200: 5).

**Table 2:** Shows a comparison of the performance of C750 and C1200 stratified by evaluation categories and readers.

Category/Reader	Reader 1	Reader 2	Reader 3	Mean
A: Liver arteries visualization	67% (*p* = 0.22)	50% (*p* = 0.87)	100% (*p* = 0.03)*	72% (*p* = 0.18)
B: Tumor Blush	100% (*p* = 0.002)*	100% (*p* = 0.007)*	100% (*p* = 0.02)*	100% (*p* < 0.001)*
C: CM uptake in the liver parenchyma	83% (*p* = 0.22)	67% (*p* = 0.18)	92% (*p* = 0.02)*	81% (*p* = 0.42)
D: Stability of catheter in CHA	92% (*p* = 0.78)	75% (*p* = 0.11)	83% (*p* = 0.37)	83% (*p* = 0.67)
Total	75% (*p* = 0.07)	67% (*p* = 0.23)	75% (*p* = 0.02)*	69% (*p* = 0.13)

The percentage of patients in which C1200 scored equal or higher than C750 on the Likert scale is shown per reader. The *p*-value of a paired *t*-test is shown in brackets. CM, contrast media; CHA, common hepatic artery.

* Indicates significance with *p* < 0.05.

Regarding tumor blush (category B), C1200 significantly outperformed C750 across all readers (100% each, mean score C1200 vs. C750: 1.3 vs. 0; 1.2 vs. 0; 0.8 vs. 0 points, *p* < 0.001; Table 2).

**Figure 2:**
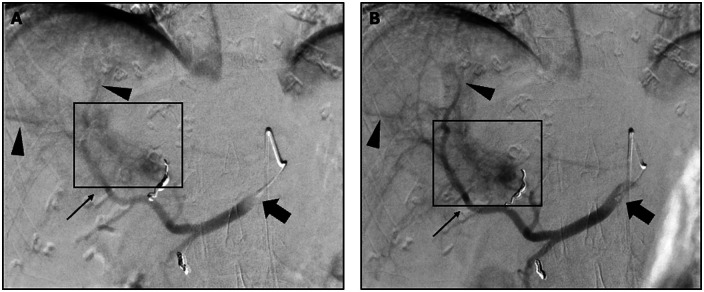
Shows arterial contrast and contrast medium distribution in central (arrows) and peripheral (arrowheads) arteries during a representative DSA using high-pressure injection with C750 **(A)** and C1200 **(B)** in the same patient. Both microcatheter tips (block arrows) were positioned comparably in the common hepatic artery. Compared to C750, C1200 demonstrates superior arterial opacification, more homogeneous contrast medium distribution, clearer tumor blush demarcation (box), and greater injection stability. DSA, digital subtraction angiography.

For liver parenchymal CM uptake (category C), C1200 was rated equal or higher than C750 in 83%, 67%, and 92% of cases (significant in reader 3, *p* = 0.02). C1200 was rated equal or higher than C750 in 92%, 75% and 83% of cases, for its stability in the CHA at the time of injection (category D). These results were not statistically significant (1: *p* = 0.78; 2: *p* = 0.11; 3: *p* = 0.37). Overall, C1200 showed equal or superior performance across all categories in 75%, 67% and 75% of cases (Figure 2; Supplementary Appendix S11).

#### 3.4.2 Quantitative image assessment

**Figure 3:**
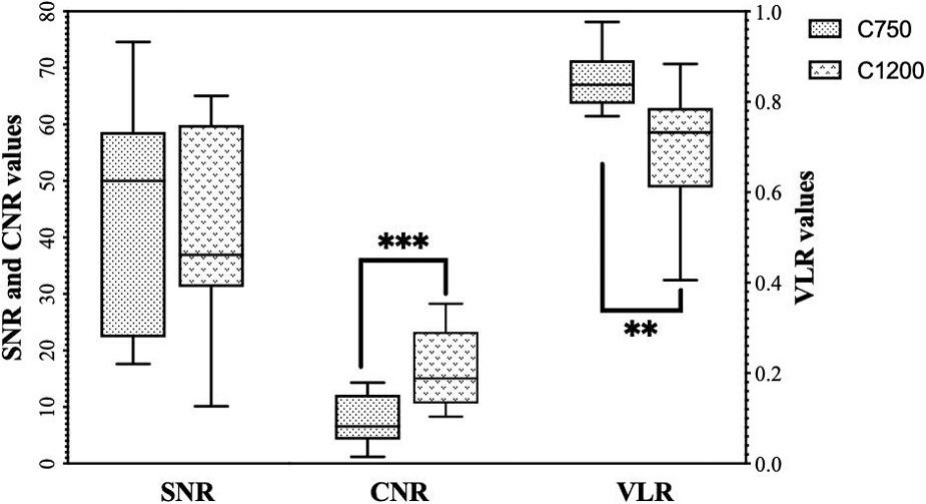
Shows a whisker plot illustrating SNR, CNR and VLR for both C1200 and C750. Whiskers indicate maximum and minimum values. Boxes extend from the 25th to 75th percentiles. The line in the middle of the box is plotted at the median. ** = *p* < 0.01, *** = *p* < 0.001, according to a Wilcoxon-Signed Rank test. SNR, signal-to-noise-ratio; CNR, contrast-to-noise-ratio; VLR, vessel-to-liver signal intensity ratio.

SNR (median C1200 vs. C750: 36.9 vs. 50.0, *p* = 0.62) and VLR (median C1200 vs. C750: 0.7 vs. 0.8, *p* = 0.001) were lower using C1200 compared to C750. CNR values were higher in C1200 (median C1200 vs. C750: 15.1 vs. 6.6, *p* < 0.001; Figure 3).

## 4 Discussion

This prospective comparative study found that higher injection pressure can improve DSA image quality during abdominal angiography using a high-pressure microcatheter. Despite variability in interrater agreement, C1200 proved superior in tumor blush visualization and maintaining stability in the CHA during injection. No significant difference was observed regarding DAP (*p* = 0.95), likely due to the small cohort. Potential radiation optimization by improved DSA image quality remains speculative and cannot be fully supported with the data provided. The questionnaire developed for this study includes previously established visualization assessments (15) and additionally evaluates CM flow and reflux, tumor to parenchymal contrast, and tumor blush. Our questionnaire thereby exceeds established analyses and provides an extensive image quality assessment specific to HCC. Quantitative analyses were performed to complement the qualitative questionnaire assessments, using parameters such as CNR, SNR, and VLR. As no validated method currently exists for DSA image quality quantification, this study adapts an established approach from CT angiography, which requires validation in future studies.

Prior studies have highlighted that DSA quality is crucial for visualizing vascular anatomy and pathology in embolization procedures (16). The improved image quality observed with higher-pressure injection is primarily attributable to the increased injection pressure itself, which not only enhances contrast distribution but also enables full contrast delivery. A pig model demonstrated faster CM injection to produce higher peak pressures, correlating with better enhancement and a narrower scan window (17). Takagi et al. showed that higher injection rates enhance contrast in wide-lumen vessels during DSA (8). However, injection rates are proportional to the pressure in laminar flow and increase with higher injection pressure. Therefore, flow rates are limited by the injection pressure tolerated by the microcatheter used.

To ensure consistent and reproducible contrast delivery across all patients, the microcatheter tip was placed in the CHA. Although larger catheters (e.g., 4F or 5F) could be used at this level, anatomical variations in hepatic artery origin and celiac trunk configuration can limit distal access. While our study focused on injection pressure during DSA, CBCT has emerged as a valuable tool for improved identification of arterial origins and enhanced image guidance (18, 19). Future studies should evaluate whether higher injection pressures can further enhance vessel visibility in CBCT and software-guided workflows.

This study has several limitations. The sample size of twelve patients is small but commonly considered sufficient for a pilot study (13). As a result of the small sample size, the data is sensitive to outliers. Microcatheter placement in the CHA and flow patterns can be affected by anatomical variants and arteriosclerosis. Furthermore, tumor blush can depend on tumor location and vascularity. However, this heterogeneity is not relevant as comparisons between the two catheters were conducted within the same patient with identical vascularity. The injector settings were established using a high-quality *ex vivo* model, which is not fully capable to replicate *in vivo* conditions such as pulsatile arterial flow, variation in vessel compliance and resistance. The moderate interrater agreement may stem from nuances in imaging, causing variability in questionnaire answers. These results may vary according to the type of contrast media and injection temperature used, thus generalizability of these findings may be reduced.

In conclusion, the findings of this prospective pilot study suggest potential benefits of standardized CM injections for DSA using higher injection pressure to enhance image quality and tumor demarcation during IATs. In turn, this could reduce risks associated with repeated CM injections and minimize procedure time. If confirmed in a larger cohort, these findings may further improve the precision of IATs through clearer visualization of tumor vasculature, enable more reliable delivery of therapeutic agents, and improve both procedural efficiency and patient outcomes if implemented into clinical practice.

## Data Availability

The datasets presented in this article are not readily available because the datasets generated and/or analysed during the current study are not publicly available due to institutional data protection regulations but are available from the corresponding author on reasonable request. Requests to access the datasets should be directed to lynn-jeanette.savic@charite.de.

## References

[1] Kamaya A Maturen KE Tye GA Liu YI Parti NN Desser TS. Hypervascular liver lesions. Semin Ultrasound CT MR. (2009) 30(5):387–407. 10.1053/j.sult.2009.06.001 19842564

[2] European Association for the Study of the Liver. EASL clinical practice guidelines: management of hepatocellular carcinoma. J Hepatol. (2018) 69(1):182–236. 10.1016/j.jhep.2018.03.019 29628281

[3] Reig M Forner A Rimola J Ferrer-Fàbrega J Burrel M Garcia-Criado A BCLC strategy for prognosis prediction and treatment recommendation: the 2022 update. J Hepatol. (2022) 76(3):681–93. 10.1016/j.jhep.2021.11.018 34801630 PMC8866082

[4] Lanza C Ascenti V Amato GV Pellegrino G Triggiani S Tintori J All you need to know about TACE: a comprehensive review of indications, techniques, efficacy, limits, and technical advancement. J Clin Med. (2025) 14(2):314. 10.3390/jcm14020314 39860320 PMC11766109

[5] Liapi E Geschwind JF. Intra-arterial therapies for hepatocellular carcinoma: where do we stand?. Ann Surg Oncol. (2010) 17(5):1234–46. 10.1245/s10434-010-0977-4 20405328

[6] Giurazza F Contegiacomo A Corvino F Rebonato A Castiglione D Palmucci S Post-traumatic intraparenchymal renal hemorrhages: correlation between CT and DSA vascular findings for superselective embolization procedures. Diagnostics. (2021) 11(7). 10.3390/diagnostics11071256 34359339 PMC8305924

[7] Bannangkoon K Hongsakul K Tubtawee T. Impact of cone-beam computed tomography with automated feeder detection software on the survival outcome of patients with hepatocellular carcinoma during treatment with conventional transarterial chemoembolization. BMC Gastroenterol. (2021) 21(1):419. 10.1186/s12876-021-02004-z 34749658 PMC8574007

[8] Takagi S Hanasaki N. Relationship between injection rate and contrast enhancement on three-dimensional digital subtraction angiography of the cerebral arteries. J Belg Soc Radiol. (2018) 102(1):76. 10.5334/jbsr.1619 30533599 PMC6284140

[9] von Elm E Altman DG Egger M Pocock SJ Gotzsche PC Vandenbroucke JP The strengthening the reporting of observational studies in epidemiology (STROBE) statement: guidelines for reporting observational studies. Lancet. (2007) 370(9596):1453–7. 10.1016/S0140-6736(07)61602-X 18064739

[10] Ren H Zhen Y Gong Z Wang C Chang Z Zheng J. Feasibility of low-dose contrast media in run-off CT angiography on dual-layer spectral detector CT. Quant Imaging Med Surg. (2021) 11(5):1796–804. 10.21037/qims-20-925 33936965 PMC8047342

[11] Agarwal A Kumar KP Madhusudhan KS. Utility of dual energy CT angiography in the evaluation of acute non-variceal gastrointestinal hemorrhage: comparison with digital subtraction angiography. Abdom Radiol. (2023) 48(6):1880–90. 10.1007/s00261-023-03864-7 36939912

[12] Kunselman AR. A brief overview of pilot studies and their sample size justification. Fertil Steril. (2024) 121(6):899–901. 10.1016/j.fertnstert.2024.01.040 38331310 PMC11128343

[13] Julious SA. Sample size of 12 per group rule of thumb for a pilot study. Pharm Stat. (2005) 4(4):287–91. 10.1002/pst.185

[14] Koo TK Li MY. A guideline of selecting and reporting intraclass correlation coefficients for reliability research. J Chiropr Med. (2016) 15(2):155–63. 10.1016/j.jcm.2016.02.012 27330520 PMC4913118

[15] Wen X Jiang X Li R Zhang J Yang P Shen B. Novel x-ray imaging technology allows substantial patient radiation reduction without image quality impairment in repetitive transarterial chemoembolization for hepatocellular carcinoma. Acad Radiol. (2015) 22(11):1361–7. 10.1016/j.acra.2015.07.010 26472122

[16] Hassan AE Wise J Burke EM Tekle WG. Visualization of flow diverter stent wall apposition during intracranial aneurysm treatment using a virtually diluted cone beam CT technique (vessel ASSIST). Neuroradiology. (2021) 63(1):125–31. 10.1007/s00234-020-02507-8 32740710

[17] Lell MM Jost G Korporaal JG Mahnken AH Flohr TG Uder M Optimizing contrast media injection protocols in state-of-the art computed tomographic angiography. Invest Radiol. (2015) 50(3):161–7. 10.1097/RLI.0000000000000119 25478743

[18] Park SJ Cho Y Lee HN Lee S Chung HH Park CH. Enhancing procedural decision making with cone beam CT in renal artery embolization. Sci Rep. (2024) 14(1):18198. 10.1038/s41598-024-69363-x 39107426 PMC11303547

[19] Rostambeigi N Crawford D Golzarian J. Benefits and advances of cone beam CT use in prostatic artery embolization: review of the literature and pictorial essay. CVIR Endovasc. (2024) 7(1):46. 10.1186/s42155-024-00459-1 38744805 PMC11093965

