# Supplementary material for: Effects of contrast medium injection pressure on angiographic image quality
Source: Front Radiol. 2026 Feb 2;5:1723413. doi: 10.3389/fradi.2025.1723413 (PMC12907394; doi:10.3389/fradi.2025.1723413)
Supplement: Supplementary file 1 [file Table1.docx]

**SUPPLEMENTARY MATERIAL**

**
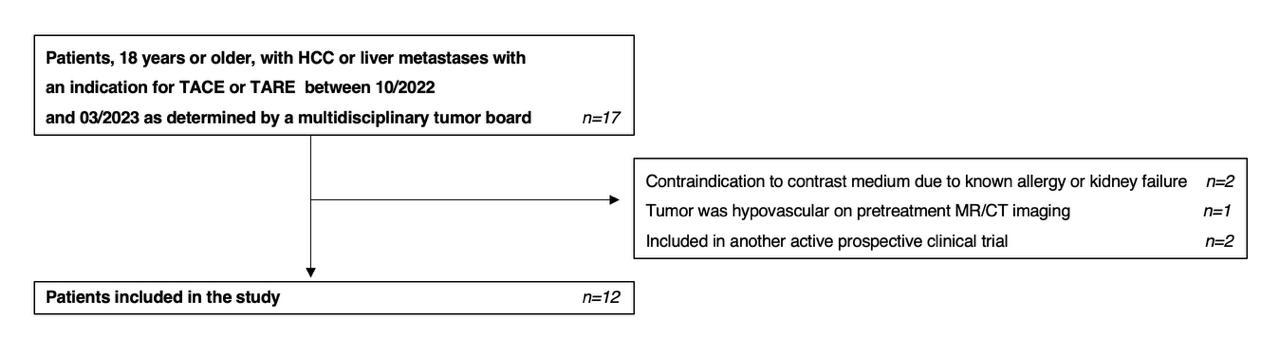
**

**S1. Figure S1** shows a flowchart illustrating the patient selection process in the study, adapted from the STROBE guidelines.

*Abbreviations: HCC: hepatocellular carcinoma; TACE: Transarterial Chemoembolization; TARE: Transarterial Radioembolization*

**S2.** The Accutron HP contrast media (CM) injector (Medtron AG, Germany) was used. The injector’s piston was connected to a FLEX120RAN high-pressure extension tube (Guerbet, France) with a P_max_ of 1200 PSI. One end of a high-pressure L306 three-way stopcock (BD, USA) with a P_max_ of 1200 PSI was connected to the extension tube. The second end of the three-way stopcock was connected to C1200 or C750. A ME780S pressure sensor (Amsys, Germany) with a P_max_ of 100 bar (1450 PSI) was connected to the piston to accurately measure the CM pressure at the microcatheter’s tip. The sensor was embedded in a 90XD metal case (Amsys, Germany), which was in turn attached at the third end of the three-way stopcock. This set-up is shown in figure 1. The injector’s piston was filled up with 100% CM Iomeprol (Iomeron® 300) (Bracco Imaging Deutschland GmbH, Germany) with a concentration of 300 mg Iodine/ml used at room temperature.

**S3.** The model is made from plastic tubes simulating the liver arteries and was filled up with water to reach a pressure of around 100-120 mmHg simulating the pressure inside the common hepatic artery (CHA).

**S4.** The pressure sensor had a baseline reading of 0.5 Volts (V). When the contrast medium (CM) flowed through the connection, the sensor detected a voltage (V), which was recorded using a multimeter connected to the sensor. The sensor's output is such that 1 Volt corresponds to 25 bars of pressure. The voltage (V) is then converted to pressure (bar) using the following formula:

$$P=\left( \left( U-0.5 \right) x 25 \right)-Patm$$

where P is the pressure in bars, U is the measured voltage (V), and Patm is the atmospheric pressure, taken as 1.01325 bar. The pressure in bars is later converted to PSI using the standard conversion factor, where 1 bar = 14.50377377 PSI.

**S5.** Specifically, qualitative imaging components (i.e. arterial contrast, tumor blush) were rated on a Likert scale or in direct comparison. Similar questions were grouped into four categories. Category A entailed questions evaluating the visualization of central and peripheral liver arteries during the DSA, the extent of CM reflux during injection, and CM flow quality as being either smooth or disrupted. Category B focused on the tumor blush, and Category C evaluated the tumor to parenchymal contrast. Category D evaluated catheter instability or dislocation during injection. Responses were converted to numerical values, with categories A, B, and C being primary endpoints and weighted more heavily (x2) for the final score calculation.

**S6.** SNR and CNR calculations were adapted from CT angiography images used by Agarwal et al (1). 15 ROIs were placed in vessels: 3 each in the CHA, left hepatic artery, right hepatic artery, segmental arteries, and subsegmental arteries. 3 ROIs were placed in liver background (non-tumor liver parenchyma without vessels) on representative DSA images for each patient.

$$SNR=\frac{mean\left( signal in vessel ROIs \right)}{standard deviation(signal in liver background ROIs)}$$

$$CNR=\frac{mean\left( signal in vessel ROIs \right)-mean(signal in liver background ROIs)}{standard deviation(signal in liver background ROIs)}$$

$$VLR=\frac{mean\left( signal in vessel ROIs \right)}{mean(signal in liver background ROIs)}$$

**S7.** **Table S7** showing the volume of contrast media applied during the DSA by the injector for each patient.

| **Patient** | **CM applied with C1200 (ml)** | **CM applied with C750 (ml)** |
| --- | --- | --- |
| 1 | 20 | 13 |
| 2 | 20 | 20 |
| 3 | 20 | 20 |
| 4 | 20 | 20 |
| 5 | 20 | 19 |
| 6 | 20 | 20 |
| 7 | 20 | 20 |
| 8 | 20 | 20 |
| 9 | 20 | 14 |
| 10 | 20 | 17 |
| 11 | 20 | 13 |
| 12 | 20 | 15 |
| **Average** | 20 | 17.7 |

*Abbreviation: CM: contrast media*

**S8. Table S8** showing the image acquisition parameters during all DSA runs for each patient.

| **Patient** | **KVP (kV)** | **X-ray tube current (mA)** | **Pulse width (ms)** | **Filter thickness (mm)** | **Table height (mm)** |
| --- | --- | --- | --- | --- | --- |
| 1 | 81 | 41.7 | 4.8 | 0.3 | 181 |
| 2 | 72.5 | 121.1 | 13.4 | 0.6 | 206.7 |
| 3 | 89 | 106.3 | 6.2 | 0.9 | 193 |
| 4 | 73 | 114 | 7.5 | 0.9 | 199.8 |
| 5 | 78 | 119.2 | 8.6 | 0.9 | 200.3 |
| 6 | 82.6 | 121.1 | 10 | 0.9 | 194.8 |
| 7 | 77 | 116.5 | 9.6 | 0.9 | 215.7 |
| 8 | 72 | 116.3 | 8 | 0.9 | 239 |
| 9 | 74 | 96.1 | 5.9 | 0.3 | 167.9 |
| 10 | 81 | 67.5 | 4.7 | 0.3 | 133.2 |
| 11 | 81 | 41.8 | 3.9 | 0.3 | 107 |
| 12 | 77 | 108.9 | 8 | 0.9 | 294 |

**S9.** **Table S9** showing the DAP for each patient as a measure of the total radiation dose and the number of images per DSA series as a measure of scan duration.

|  | **C1200** | | **C750** | |
| --- | --- | --- | --- | --- |
| **Patient** | **Total DAP (µGym^2^)** | **Number of images per DSA series** | **Total DAP (µGym^2^)** | **Number of images per DSA series** |
| 1 | 599.51 | 25 | 338.28 | 18 |
| 2 | 305.98 | 23 | 292.79 | 22 |
| 3 | 640.25 | 25 | 691.81 | 27 |
| 4 | 207.31 | 20 | 228.08 | 22 |
| 5 | 460.39 | 21 | 498.55 | 21 |
| 6 | 469.96 | 15 | 846.44 | 27 |
| 7 | 506.89 | 20 | 456.29 | 18 |
| 8 | 490.58 | 16 | 548.16 | 20 |
| 9 | 36.68 | 21 | 37.02 | 20 |
| 10 | 339.6 | 26 | 260.64 | 20 |
| 11 | 79.11 | 27 | 69.91 | 24 |
| 12 | 326.41 | 23 | 229.81 | 21 |
| **Average** | 371.9 | 21.8 | 374.8 | 21.7 |

*Abbreviation: DAP: dose area product*

*
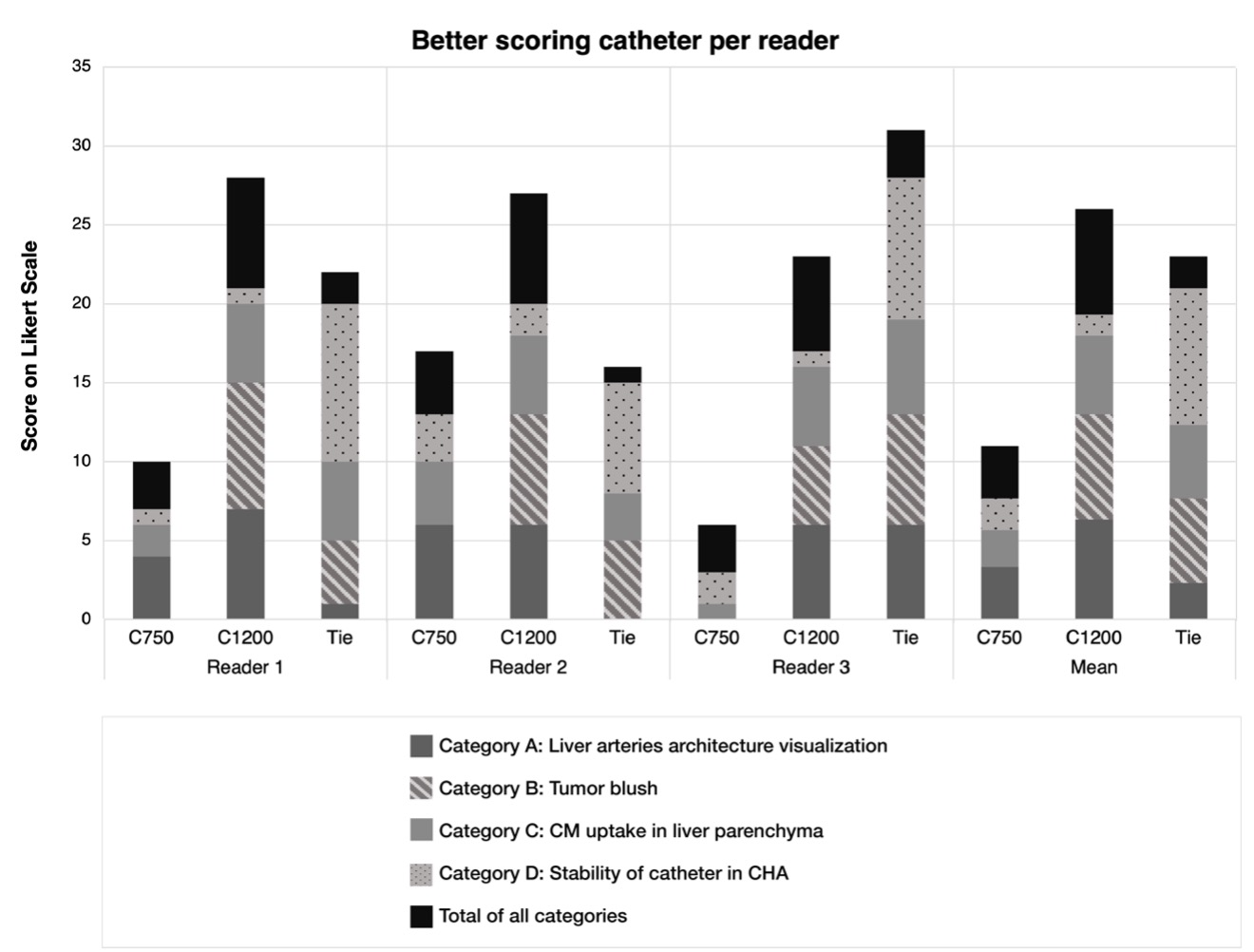
*

**S10. Figure S10** shows the number of times when C750 or C1200 microcatheter scored higher in each category, stratified by reader.


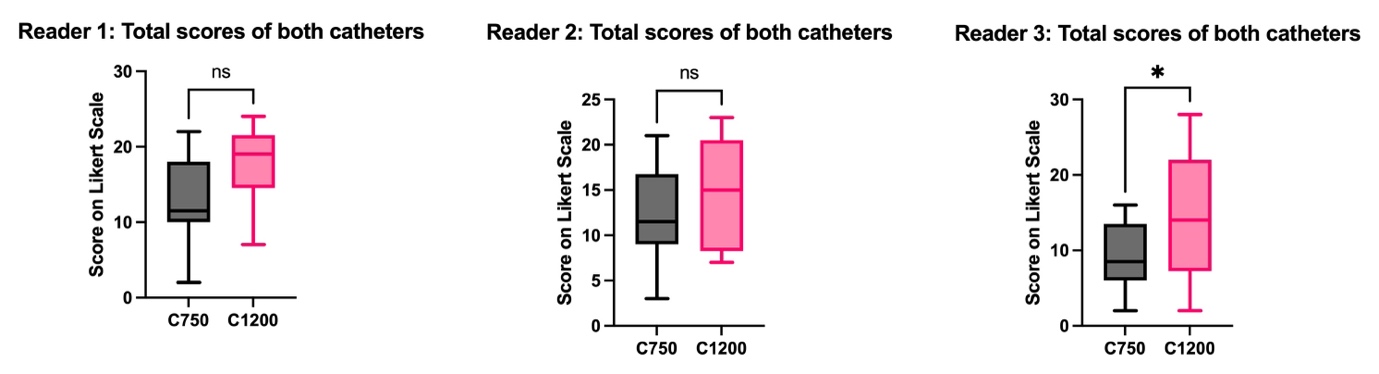


**S11**. **Figure S11** showing a comparison of the total Likert scale scores of both catheters, stratified by reader.

* Indicates a significant paired t-test, p<0.05.

**SUPPLEMENTARY REFERNCES**

1. Agarwal A, Kumar KP, Madhusudhan KS. Utility of dual energy CT angiography in the evaluation of acute non-variceal gastrointestinal hemorrhage: comparison with digital subtraction angiography. Abdom Radiol (NY). 2023;48(6):1880-90.
